# *In-silico* analysis of water and carbon relations under stress conditions. A multi-scale perspective centered on fruit

**DOI:** 10.3389/fpls.2013.00495

**Published:** 2013-12-09

**Authors:** Valentina Baldazzi, Amélie Pinet, Gilles Vercambre, Camille Bénard, Benoît Biais, Michel Génard

**Affiliations:** ^1^Institut National de la Recherche Agronomique, UR 1115 Plantes et Systèmes de Culture HorticolesAvignon, France; ^2^Institut National de la Recherche Agronomique, UR 1332 Biologie du Fruit et PathologieVillenave d'Ornon, France

**Keywords:** model, development, cell division, cell expansion, symplasm, plant architecture, tomato, stress

## Abstract

Fruit development, from its early stages, is the result of a complex network of interacting processes, on different scales. These include cell division, cell expansion but also nutrient transport from the plant, and exchanges with the environment. In the presence of nutrient limitation, in particular, the plant reacts as a whole, by modifying its architecture, metabolism, and reproductive strategy, determining the resources available for fruit development, which in turn affects the overall source-sink balance of the system. Here, we present an integrated model of tomato that explicitly accounts for early developmental changes (from cell division to harvest), and use it to investigate the impact of water deficit and carbon limitation on nutrient fluxes and fruit growth, in both dry and fresh mass. Variability in fruit response is analyzed on two different scales: among trusses at plant level, and within cell populations at fruit level. Results show that the effect of stress on individual cells strongly depends on their age, size, and uptake capabilities, and that the timing of stress application, together with the fruit position on the plant, is crucial in determining the final phenotypic outcome. Water deficit and carbon depletion impacted either source size, source activity, or sink strength with contrasted effects on fruit growth. An important prediction of the model is the major role of symplasmic transport of carbon in the early stage of fruit development, as a catalyst for cell and fruit growth.

## Introduction

Understanding the mechanisms underpinning fruit development from its early stages is of primary importance for biology and agronomy, and an open challenge for plant mathematical modeling. Indeed, the early stages are highly sensitive to biotic and abiotic stresses, with important consequences on fruit development and yield (Baldet et al., 2002; Ruan et al., 2012).

During the first phase of its development, the fruit undergoes a period of intense cell division, in which the number of cells is established, followed by a phase of rapid cell expansion. This passage is accompanied by important changes in cell metabolism, cell wall mechanical properties as well as in the mechanisms governing carbon inflow, through the symplasmic and apoplasmic pathways. On the plant side, fruit set determines a new sink for carbon, whose strength in turn depends on nutrient availabilities from the plant itself.

In the presence of nutrient limitation or a fluctuating environment, the plant reacts as a whole, by modifying its architecture, metabolism and reproductive strategy, in a complex network of interacting mechanisms (Granier and Tardieu, 2009). The consequences of stress depend on plant sources, sink load, and developmental stage at the time of stress, resulting in a range of possible phenotypic outcomes of variable significance for agronomical purposes.

Accordingly, a comprehensive analysis of fruit set and development, in relation to carbon and water availability, should take into account all the different organizational levels and the way they interact together in order to assure a common response against stressful conditions (Baldazzi et al., 2012b). Because of system complexity, however, most models have mainly focused on a single level of description, with occasional “glances” toward others.

Among the most advanced examples of integrated models, the L-PEACH model (Allen et al., 2005) describes the growth of a peach tree, as a function of environmental conditions and horticultural practices. In its current version, it combines a detailed description of the 3D plant architecture with a mechanistic representation of selected physiological functions, including nutrient assimilation, transport, and allocation (Da Silva et al., 2011). However, fruit sink growth, is described as a simple function of local carbon and water availabilities, regardless of fruit developmental stages and metabolism.

A better description of fruit growth is offered by the QUALITREE model (Lescourret et al., 2010), for the peach tree again: it explicitly accounts for fruit fluxes and quality build-up, according to the virtual fruit model by Génard and colleagues (Fishman and Génard, 1998; Lescourret and Génard, 2005). As for the tomato, the biophysical description of the virtual fruit model has recently been combined with a mechanistic model of water transport and storage, which includes variations in stem diameter and their effect on xylem and phloem water potential (De Swaef et al., 2013), but with no interaction with carbon acquisition and metabolism.

On a finer scale, fruit cellular composition in terms of cell metabolic and mechanical properties adds an additional level of complexity, contributing to the observed variability in stress tolerance, especially during the early developmental phase.

Although cell division and cycle progression has largely been studied at the molecular level, its interaction with cell expansion remains poorly understood, and is the topic of an ongoing debate (Fleming, 2006; John and Qi, 2008; Harashima and Schnittger, 2010; Gonzalez et al., 2012). As a consequence, the cell division phase has rarely been integrated into models of organ development (Dupuy et al., 2010; Roeder et al., 2010; Asl et al., 2011). Moreover, the effect of environmental factors on cell division and growth is often left implicit, making these models less amenable to an *in-silico* exploration of stress effect on individual physiological processes (Granier and Tardieu, 1999; Granier et al., 2000; Tardieu et al., 2011). An exception is the short-term response to temperature that has been modeled for different physiological processes, including cell division, based on thermodynamic principles (Parent et al., 2010). A model integrating cell division, expansion, and endoreduplication in the tomato has recently put forward by Fanwoua et al. (2013). Whereas the progression of cell division is described as a function of thermal time, cell growth is modeled following a simple source-sink approach, thus neglecting the effect of water availability and transport processes on fruit expansion.

Here we present a tomato model that explicitly accounts for early developmental changes (from cell division to harvest) and use it to virtually investigate the impact of water and carbon limitations on nutrient fluxes, and the resulting effect on fruit development and growth, in both dry and fresh mass.

Variability in fruit response is analyzed on two different scales. At the organ level, variability among cells is considered by coupling the growth model to an existing cell division model, providing the time-course for cell proliferation and its progressive slow-down during fruit development. The effect of stress on individual cells is analyzed through the model as a function of their age, size, transport properties, and timing of stress application. At the plant level, plausible plant architecture mock-ups are used to estimate resource acquisition (carbon) and water transpiration distribution under different environmental conditions. Plasticity between growth in dimension and structural growth is introduced, especially through variation in the specific leaf area. Carbohydrate storage and mobilization are integrated as well, and photosynthesis is made dependent on leaf carbon and water status so that the effects of stress on water and carbon availability are the output of the model. The consequences of water deficit and carbon limitation on fruit growth are analyzed through the model as a function of fruit position, at developmental stage.

## Materials and methods

### The plant model

#### Plant architecture: topology, organogenesis, and morphogenesis

The plant architecture was considered as a collection of elementary units called phytomers. Each phytomer is composed of one stem internode and one composite leaf (vegetative phytomer) or one truss (generative phytomer). Each leaf consists of a leaf rachis, with pairs of lateral leaflets, and one terminal leaflet. Each truss is composed of a rachis, with 2 pairs of lateral fruits and one terminal fruit, as fruit trusses were thinned to 5 fruits per truss. The first generative phytomer appeared on the 9th phytomer, and was then produced every three phytomers. Only the main stem is considered, as side shoots are classically removed.

The production of phytomers was related to the phyllochron and thermal time. The phyllochron was constant during plant growth. After its emergence and a given lag time, each organ started its growth (Najla et al., 2009). Leaf and stem internode elongations were characterized by two parameters: the elongation rate and the elongation duration, assuming that growth is linear. The duration of internode elongation was assumed to be equal to that of leaf elongation. Leaf surface was estimated using an allometric relationship between its length, width, and rank. Leaf rachis elongation was estimated using the leaf elongation and the proportion of rachis over the whole leaf length. The radial growth of the leaf rachis was estimated using an empirical linear relationship between its cross-sectional area and the leaf area. The radial growth of stem internodes was described using a pipe model (see Najla et al., 2009, for a full description). The plant architecture model leads to organ dimension rather than dry mass; leaf dry mass was therefore calculated using the specific leaf area (SLA, cm^2^.g^−1^) and leaf-age variations. Next, an allometric relationship based on leaf dry mass leads to the estimation of the dry masses of stem internodes and leaf rachis (rachis and internode fraction). For the root system, an allometric relationship links its dry mass to the vegetative aboveground biomass (Jones et al., 1991).

#### Resource acquisition and availability

**Figure 1:**
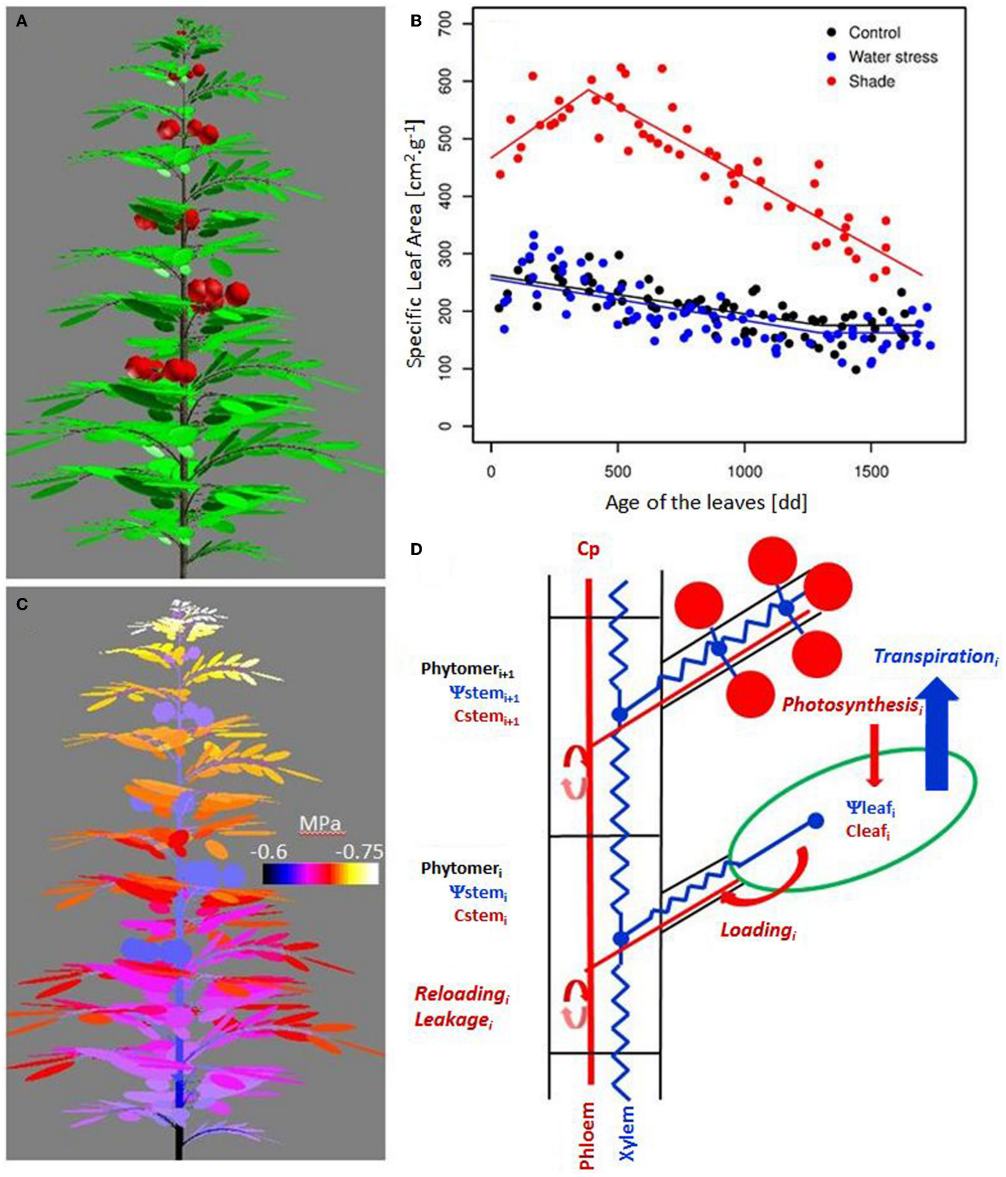
**Simulated architecture of a tomato plant (100 days old) using the dynamic 3D plant model (A), variation of the minimal water potential within the plant (C), variation of the Specific Leaf Area (SLA) with leaf age for the treatments (B), and schematic representation of the resource acquisition modeling and their transfer to the plant (D)**. Leaves are composed of different leaflets, which have similar incident radiation, transpiration, and photosynthesis. Water transfer is modeled according to the axial conductance and the distribution of the transpiration within the plant. Carbohydrate availability is assumed to be uniform in the phloem. Carbohydrate loading at leaf, carbohydrate reloading, and leakage along the path all lead to variations in storage, and therefore in carbohydrate concentration in the organ.

***Estimation of the photosynthesis and carbon availability***. The plant 3D mock-up (Figure 1A) is used to estimate radiation interception through the canopy. The canopy is composed of infinite rows of identical plants with a specified plant density. Photosynthesis is calculated on leaf scale. The radiation model enables the calculation of radiation for each plant leaf, encompassing its direct and diffuse components (De Pury and Farquhar, 1997) with sky irradiance distribution calculated from sky conditions, from clear to overcast according to a cloudless index (Igawa et al., 2004). The basic assumptions of radiation interception are: (i) the beam traveling through the canopy is attenuated according to Beer's law, (ii) the foliage is uniformly distributed within the space volume occupied by the canopy, and (iii) the leaf angle distribution is spherical. The extinction coefficient is linked to leaf inclination distribution and beam/diffuse directions (Wang et al., 2007). Dealing with scattering, the theory for horizontal canopies (Norman and Jarvis, 1975) is applied to the tomato canopy following the assumption and procedure proposed by Norman and Welles (1983). Incident radiation was estimated for each leaf, according to the position of the leaf within the canopy, and hence to the calculated path-length of the radiation. The photosynthesis model is scaled up from the biochemical model of leaf photosynthesis (Farquhar et al., 1980), using parameters estimated for the tomato (Sarlikioti et al., 2011), and by taking into account sunlit and sun-shaded leaves.

The carbohydrates originating from photosynthesis are stored in the leaf and then loaded into the phloem (Figure 1D). No transitory compounds have been introduced in the model at this stage (starch). In the tomato, sucrose enters the sieve element-companion cell complexes via an active loading fueled by H+-ATPases (DeWitt and Sussman, 1995) which was modeled like Michaelis–Menten kinetics:

(1) $${\text{Loading}}_{i}=\text{Leaf }{\text{Area}}_{i}\;V\underset{\text{leaf}}{\max}\frac{{\text{Cleaf}}_{i}}{Km_{\text{leaf}}+{\text{Cleaf}}_{i}}$$

The Cleaf carbohydrate concentration is calculated for each leaf and results from the balance between carbon intake by photosynthesis and export by phloem loading:

(2) $${\text{Cleaf}}_{i}(t)={\text{Cleaf}}_{i}(t-1)+\frac{{\text{Photosynthesis}}_{i}-{\text{Loading}}_{i}}{\text{Fresh }{\text{Mass}}_{i}}$$

Following phloem loading, the sucrose is translocated from sources to sink through the phloem network. Leakage and reloading of carbohydrates occur simultaneously along the phloem pathway. This leakage-retrieval mechanism is based on a balance between the leakage of carbohydrates from the phloem into the surrounding apoplast, and their subsequent retrieval (Van Bel, 2003). In the model, such processes occur for each internode and rachis. The leakage mechanism is associated to a diffusion process, and is modeled by a linear function of the carbohydrate phloem concentration, since the apoplastic concentration is very low (Patrick et al., 2001):

(3) $${\text{Leakage}}_{i}=\text{Fresh }{\text{Mass}}_{i}\;kC_{\text{phloem}}$$

The retrieval process is dependent on sucrose carriers, and is therefore sensitive to apoplastic carbohydrate concentration. Such a leakage-retrieval mechanism is important for short- and long-term buffering (Van Bel, 2003). This process is formalized by Michaelis–Menten kinetics (Patrick et al., 2001), relating carbohydrate retrieval to the carbohydrate concentration in each storage compartment and to its fresh mass:

(4) $${\text{ReLoading}}_{i}=\text{Fresh }{\text{Mass}}_{i}\;{\text{Vmax}}_{\text{storage}}\frac{C{\text{storage}}_{i}}{Km_{\text{storage}}+C{\text{storage}}_{i}}$$

Carbohydrate concentration is calculated for each storage compartment. It results from the balance between phloem leakage and carbohydrate retrieval and the fresh mass of the considered organ:

(5) $${\text{Cstorage}}_{i}(t)={\text{Cstorage}}_{i}(t-1)+\frac{\left({\text{Leakage}}_{i}-{\text{Reloading}}_{i}\right)}{{\text{FreshMass}}_{i}}$$

The modeled vegetative architecture (dimension and dry mass) is used to estimate the vegetative sink. The carbohydrate used for maintenance respiration is estimated through a maintenance respiration rate and a Q10 law to incorporate the effect of temperature (Jones et al., 1991). The growth cost, growth respiration and dry biomass accumulation, are estimated using a growth efficiency parameter accounting for growth respiration, following TOMGRO formalism (Jones et al., 1991):

(6) $$\begin{array}{l} \text{Vegetative Sink}=\sum_{i}\text{Growth}\text{Efficiency }\frac{d\text{Dry}{\text{Mass}}_{i}}{dt}\\ \;+\;Q_{10}(Temp)\text{Dry}{\text{Mass}}_{i} \end{array}$$

Fruit growth is modeled using the fruit model (see infra), assuming that the fruit mesocarp behaves as a single cell. The boundary conditions for each fruit are the xylem water potential (see infra) and the carbohydrate phloem concentration at the fruit insertion point. The carbohydrate phloem concentration is assumed to be uniform throughout the plant, whereas the xylem water potential is variable depending on the fruit location within the hydraulic plant architecture. Each fruit has its own driving variable (water potential, dry and fresh mass) but the set of parameters is identical whatever the fruit location and treatment. Therefore, the sink strength for the entire fruit collection could be written as:

(7) $$\text{Reproductive Sink}=\sum_{i}\text{Fruit}\text{ Model}\left(C_{\text{phloem}},\;\psi x_{i}\right)$$

Phloem carbohydrate concentration is calculated in order to balance carbohydrate sources (loading, balance between reloading and leakage) and sinks (vegetative and reproductive):

(8) $$\begin{array}{l} \text{Vegetative Sink}+\text{Reproductive Sink}\\ =\sum_{i}\left({\text{Loading}}_{i}-\;{\text{Leakage}}_{i}+{\text{ReLoading}}_{i}\right) \end{array}$$

***Estimation of the transpiration and water availability***. The radiation model has been coupled with an energy balance model. For each leaf, the radiation balance was split according to the three wavebands [Photosynthetically Active Radiation (PAR), Near Infrared Radiation (NIR), and Thermal Infrared Radiation (TIR)]. In the TIR, incident radiation is related to sky, soil, and leaf emissivities and their respective temperatures. The net radiation (*Rn_i_*) can therefore be written as:

(9) $$Rn_{i}=PAR_{i}+NIR_{i}+TIR_{i}-2\sigma T\;{\text{leaf}}_{i}^{4}A_{i}$$

where the last term accounts for emitted radiation of the considered leaf according to the Stefan–Boltzmann equation, taking into account its temperature (Tleaf), surface (Ai), and the Stefan–Bolzmann constant (σ). The energy balance can be written as follows:

(10) $$Rn_{i}=S_{i}+\lambda {\text{E}}_{i}$$

where *S_i_* and λ*E*_*i*_ are the sensible and latent heat fluxes, respectively. The sensible and latent fluxes are computed according to the leaf boundary and stomatal conductances (Sinoquet et al., 2001). Leaf boundary conductance encompasses a free component associated to the difference in leaf and air temperatures and a forced component related to wind speed around the leaf (Daudet et al., 1999). Stomatal conductance is expressed according to the multiplicative and empirical model of Jarvis (1976):

(11) $$g_{s}=g_{s\max}f_{1}(PAR)f_{2}(VPD)f_{3}\left(T_{\text{leaf}}\right)f_{4}\left(\psi_{\text{leaf}}\right)$$

Accordingly, the stomatal conductance is related to a maximal stomatal conductance decreased by reduction functions associated with (1) the incident PAR radiation on the leaf, (2) vapor pressure deficit (VPD), (3) leaf temperature, and (4) water potential (ψ_leaf_). By solving the energy balance amounts (Equation 9 and 10), a surface temperature is estimated where gained heat fluxes are balanced out by lost ones. The model separates the energy balance of sunlit and shaded areas, as differences in surface temperatures can be expected (Sinoquet et al., 2001). The radiative balance is sensitive to leaf temperature through the emitted thermal radiation, just as the energy balance is sensitive to leaf temperature and leaf water potential through boundary and stomatal conductances. Consequently, an iterative process is needed in order to correctly estimate first leaf temperature, then leaf transpiration and finally water potential distribution within the plant.

The three-dimensional plant architecture is linked with laws describing water flow in axes. Water flow through an axis is characterized by its hydraulic conductivity (m^4^ s^−1^MPa^−1^) and by the water potential drop along the axis, as shown Figure 1C (Fiscus, 1975). Hydraulic conductivity is assumed to vary according to axis diameter (Tyree, 1988). The plant hydraulic architecture is discretized at the phytomer level. The boundary condition is imposed at the root collar through diurnal variation in the xylem water potential.

#### The fruit model

An integrated model of tomato fruit development growth has been developed and explicitly accounts for the early phase of fruit development, coupling cell proliferation, and expansion phases.

***Expansion model***. Cell expansion is related to processes driving the water and solute fluxes into the expanding fruit tissues, including mechanisms for xylem and phloem unloading, carbon uptake and regulation of wall synthesis, and bio-rheological properties during fruit development (Cosgrove, 1993; Boyer and Silk, 2004).

A few existing models try to integrate such a complexity, assuming that the fruit mesocarp behaves as a single compartment, separated from xylem or phloem tissue by a membrane (Fishman and Génard, 1998; Liu et al., 2007). Water and solute flows across this membrane are described by thermodynamic equations, involving differences in hydrostatic and osmotic pressures on both sides of the membrane, and properties of the membrane toward water (hydraulic conductivity) and solutes (reflection coefficient and permeability). The resulting change in water content over time is calculated as the algebraic sum of xylem and phloem (*Up*) influx minus the water outflow due to fruit transpiration. The rate of dry mass change is calculated as the total carbon uptake (*Us*) minus the carbon loss by respiration. As detailed in the original models, the total uptake of carbon from phloem is the sum of contributions due to mass flow through plasmodesmata, passive diffusion and active carbon transport

(12) $$\begin{array}{l} U_{s}=\underset{\text{massflow}}{\underset{︸}{\left(1-\sigma_{p}\right)C_{s}U_{p}}}+\underset{\text{Passive}}{\underset{︸}{A_{p}p_{s}\left(C_{p}-C_{s}\right)}}\\ \;+\;\underset{\text{Active}}{\underset{︸}{\frac{s\nu_{m}C_{p}}{\left(K_{m}+C_{p}\right)\left(1+\exp(t-t^{*})/\tau_{a}\right)}}}, \end{array}$$

with

$$C_{s}=\frac{C_{f}+C_{p}}{2}$$

where s is the cell dry mass, *v_m_* is the maximum uptake rate (gC gDW^−1^ h^−1^), *K_m_* is the Michaelis constant, *C_p_* and *C_f_* are the sugar concentrations in the phloem and in the fruit, respectively (g g^−1^). *p_s_* is the solute permeability coefficient (g cm^−2^ h^−1^). The second term of the denominator of the active carbon uptake accounts for an inhibitory effect which increases with time (*t*, hour, *t* = 0 at anthesis) on a timescale τ_*a*_, since time t^*^ (Liu et al., 2007). The parameter σ_p_ represents the phloem reflection coefficient and accounts for different pathways of sugar transport from phloem to sink cells. In the tomato, like in other species, sugar transport switches from the symplasmic to the apoplasmic pathway during fruit development (Damon et al., 1988; Ruan and Patrick, 1995). The carbohydrate uptake by fruit photosynthesis during early developmental stages is not considered in this first version of the model.

Fruit expansion is taken into account by iteratively solving the Lockhart equation (Lockhart, 1965) at a time-step of 1 h:

(13) $$\frac{dV}{dt}=V\phi \left(P_{f}-Y\right)$$

where *V* is the volume of the fruit, Φ is the coefficient describing the extensibility of the cell wall and *Y* is the threshold value that the hydrostatic pressure of the fruit has to exceed before irreversible expansion occurs.

Notice that the effect of plant status and environmental conditions (temperature, air humidity) on fruit growth is included in the model, via their effect on fruit fluxes and metabolism. Developmental control is also accounted for, through the presence of specific age-dependent functions, able to modulate water and carbon fluxes according to fruit stages.

In the case of the tomato fruit, in particular, the Liu et al. (2007) model already accounts for the above-mentioned switch from symplasmic to apoplasmic pathway for carbon transport and for a progressive decrease in cell wall extensibility during fruit development (Buntemeyer et al., 1998; Proseus et al., 1999). Such a model, however, was originally developed to describe the fruit expansion phase, once cell division is almost over. The application of this model to the early phase of fruit development requires the addition of a few specific mechanisms that play a significant role during the first days of fruit life.

1. After pollination, the fruit undergoes a phase of intense divisions in which the number of cells rapidly increases. As a consequence, a large fraction of the incoming carbon is converted into cell wall, thus reducing the quantity of soluble sugars available for the regulation of osmotic pressure. In the Liu et al. (2007) model a single parameter, ssrat, describes the fraction of dry mass converted into soluble sugars. Assumed constant in the original formulation, ssrat has now been modified into a saturating function of time, recovering a constant value 10–15 days after anthesis:
  (14) $$\text{ssrat}=\text{bssrat}\left(1-e^{\left(-\text{assrat }t\right)}\right)+{\text{ssrat}}_{0}$$
2. The first weeks of tomato fruit development are accompanied by rapid changes in cuticle synthesis and composition, with important consequences on fruit exchanges and responses to abiotic and biotic environment (Leide et al., 2007). In particular, a reduced wax layer in early stages results in a high skin conductance and increased water loss by transpiration (Lee, 1990). A progressive reduction in skin conductance is then observed until a plateau is reached after 10–20 DAA, depending on the fruit genotype. Accordingly, the parameter, describing fruit conductance per unit surface, has been modified as:
  (15) $$\rho =\{\begin{array}{cc} \rho_{0}+\rho_{1} & t<\text{anthesis}\\ \rho_{0}e^{\left(-k_{p}(t-t_{0})\right)}+\rho_{1} & t>\text{anthesis} \end{array}$$

assuming ρ constant before anthesis.

***Cell division model***. A deterministic model is used to predict the dynamics of cell numbers in the tomato fruit pericarp (Bertin et al., 2003) as well as the transition from cell division to cell expansion. The model considers a succession of regular division steps together with a progressive decline of the proliferative activity. So the early phase of intensive divisions is followed by a phase where both division and expansion occur simultaneously until the proliferative activity tends to zero and all cells expand. The decrease in cell proliferative activity starts before anthesis as observed in young fruit (Bertin et al., 2007) and it has been estimated here to be about 6 days before anthesis. The model includes 3 parameters: the time at which the proliferative activity starts to decline, its decrease rate, and the duration of the cell cycle. All these parameters are supposed to be independent from environmental conditions.

***Integrated cell division and expansion model***. The integrated model starts at the end of the pure division phase, once the proliferating activity of the cells declines and the expansion phase begins (Baldazzi et al., 2012a). The fruit is described as a collection of cell populations, each one having a specific age and volume. The number, age (birth date), and physiological state (proliferating or growing cells) of each population is predicted by the cell division model. Accordingly, the fraction of proliferating cells declines during development and cell division stops completely at the onset of fruit maturity. At the beginning of the simulation, all cells are supposed to be in the proliferating state. The initial number of cells is *N*(0) = *n* and the initial mass of the fruit is *V*(0) = *nm0* = *n*(*w0* + *s0*), where *w0* and *s0* are initial cell water and dry mass, respectively. At any time, cells leaving the proliferating phase are assumed to skip the next division cycle and start to grow from a doubled mass 2*m0* according to the expansion model and current environmental conditions. The model assumes that all cells have equal access to external resources (no competition among cells).

Most time-dependent functions accounting for developmental regulation of growth are calculated as a function of *cell age*. As a consequence, two cells with the same age but born at different fruit stages will behave similarly concerning carbon metabolism, transport, and wall mechanical properties. The only exception is the switch from symplasmic to apoplastic pathway for phloem unloading (parameter) which is assumed to depend on fruit age, as observed by Zambrinsky and colleagues (Crawford and Zambryski, 2001; Zambryski, 2004). Transpiration is computed at the fruit level, as a function of fruit surface and cuticular conductance, assuming that all cells contribute in proportion to their water content.

**Figure 2:**
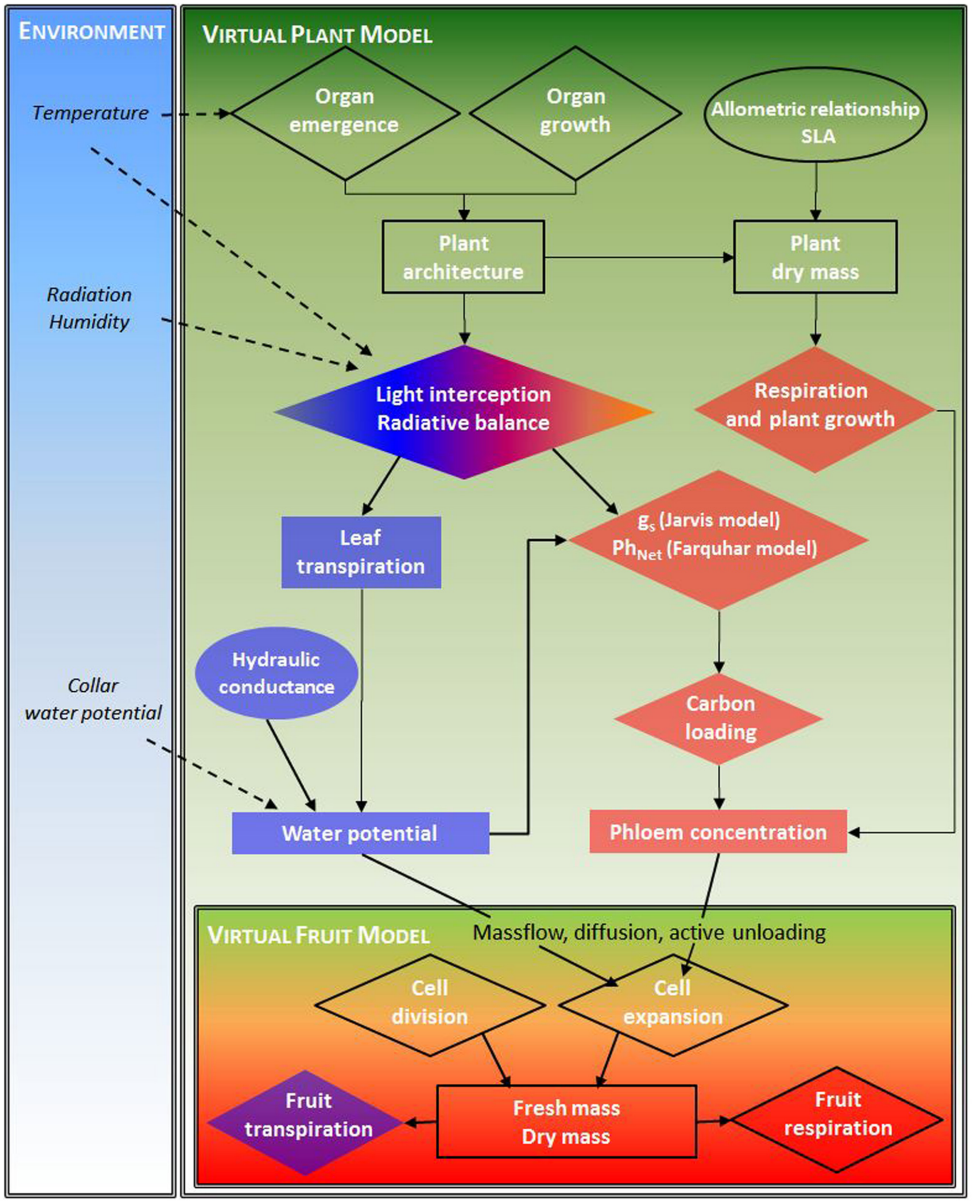
**Model flowchart depicting the different processes (diamond), variables (rectangle) at the 2 levels (fruit, plant) and their interactions (arrows) for water and carbon resources, and the environmental boundary condition**.

A flowchart of the models, depicting the different processes, variables at the 2 levels (fruit, plant) and the integration of the virtual fruit model is shown Figure 2.

#### Model parameterization and in-silico simulations

The plants were submitted to three virtual treatments: control, water deficit, and shade.

The xylem water potential driving the plant model was imposed at the tomato collar using classical pre-dawn and minimal stem water potential measured throughout the season. Pre-dawn water potential was similar whatever the treatments (−0.05 MPa). The minimal collar water potential was similar for the control and shade treatments (−0.65 MPa), and decreased by 0.1 MPa for the water deficit treatment. The shading treatment consists in a 70% decrease in the incident radiation. The treatment was applied 50 days after sowing (DAS). Data for air temperature and humidity corresponding to a natural greenhouse climatic scenario was provided to the model.

For the integrated fruit model, the phloem sugar concentration is assumed to vary daily between 0.15 and 0.35 M whereas stem water potential oscillates between −0.05 and −0.6 MPa, in standard conditions. Water deficit is simulated by decreasing stem water potential, hence leading to its variation from −0.2 to −0.8 MPa, whereas carbon depletion is virtually applied by reducing the phloem carbon concentration by 50%. Both carbon and water deficit are applied for 24 h only, at three different developmental stages, in order to neglect the effect of stress on cell division progression. The temperature and relative humidity were those used for the plant model.

Growth in dimension, allometric relationships, SLA variation with age under water deficit and shade were estimated from direct measurements. Loading, leakage, and reloading parameters were assumed to be constant whatever the organ age and the treatment. Values were taken from literature (Daudet et al., 2002; Borstlap and Schuurmans, 2004; De Schepper and Steppe, 2010).

Most fruit parameter values were taken from the original models (Bertin et al., 2003; Liu et al., 2007). In the case of the integrated cell division-expansion model, time-dependent functions were partially re-parameterized to account for differences between cell and fruit developmental time.

## Results

### Plant growth, resource availabilities, and fruit growth under standard conditions

The integrated plant/fruit model makes it possible to investigate the dynamics of both plant and fruit development, as a function of environmental variables and resource availability, from sowing to fruit maturity.

**Figure 3:**
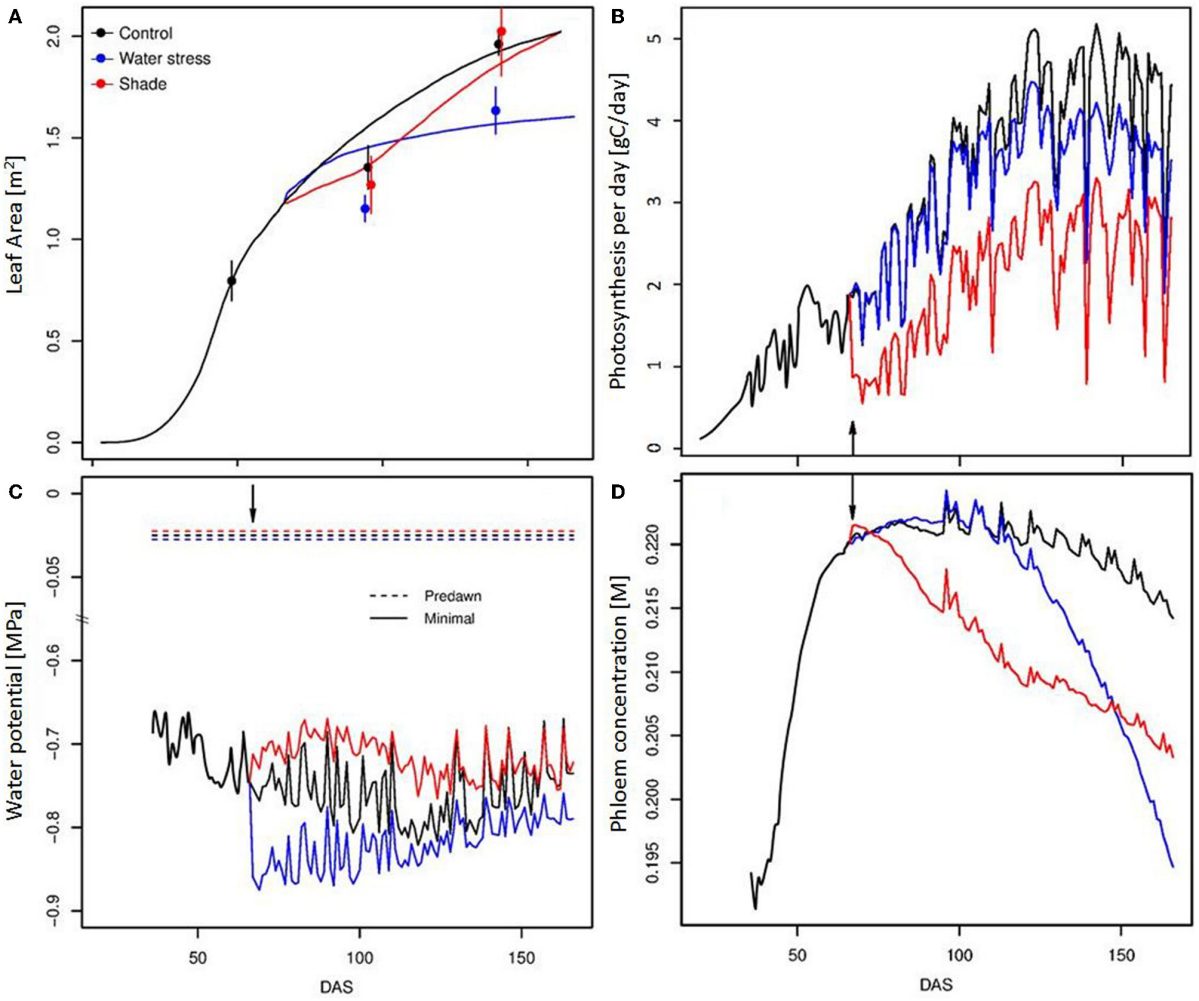
**Temporal evolution (DAS, Days After Sowing) of the leaf surface (A), of the daily plant photosynthesis (B), predawn and minimal mean stem water potentials (C) and mean daily phloem concentration (D) for the 3 modalities (control, water deficit, and shade treatment)**. The lines correspond to simulation output, and the points to experimental observations. The arrow indicates treatment start.

Vegetative plant growth was correctly simulated for the different treatments, as illustrated by the evolution of the leaf area throughout the season (Figure 3A). A large vegetative growth is observed during the young stage, until the emergence of the first truss. Vegetative growth is then reduced. The simulated as well as observed SLA, shows a gradual decline with leaf age down to a plateau (Figure 1B). During the young stage, the leaves are the main organ of the tomato plant. Then, with trusses regularly emerging, the fruits progressively become the largest compartment, representing more than 70% of the total plant dry mass from 100 days after sowing. Plant photosynthesis as well as plant transpiration, increase with the leaf area development, then reach a plateau (Figure 3B). From this moment, the plant is composed of a generally constant number of leaves and fruits. Indeed, the plant continuously grows on its apical part, while the older leaves are removed before complete senescence, and the mature fruits are harvested on its basal part. At the end of the season, daily plant photosynthesis stabilizes and eventually decreases in relation to the decrease in incident radiation, and to a progressive reduction in the final leaf dimension with leaf rank. Fluctuations in incident radiation also explain the large variations predicted in photosynthesis on a daily basis. Plant water potential can be predicted along the plant structure by taking into account the hydraulic architecture and the distribution of the transpiration within the plant, as shown in Figure 1C. Differences of around 0.1 MPa occur between the xylem water potential at the fruit insertion point depending on their location within the architecture (bottom or top of the plant, and proximal or distal position along the truss). The mean minimal plant water potential ranges between −0.7 and −0.8 MPa throughout the season (Figure 3C). Lower water potentials are observed during the summer (ranging from 60 to 120 DAS), when the leaf area is completely developed and the climatic demand is maximal. Phloem concentration only varies in a limited range. Short term variations are large during the young plant stage (data not shown), when the carbohydrate buffering by storage is low. A global trend with a maximal concentration during the summer is observed, followed by a progressive decline (Figure 3D). The spikes in phloem concentration result from the regular removal of older leaves. Since the older leaves are sinks for carbon, i.e., leaf respiration is greater than its photosynthesis, their removal leads to an increase in the source/sink ratio, and therefore in phloem concentration.

**Figure 4:**
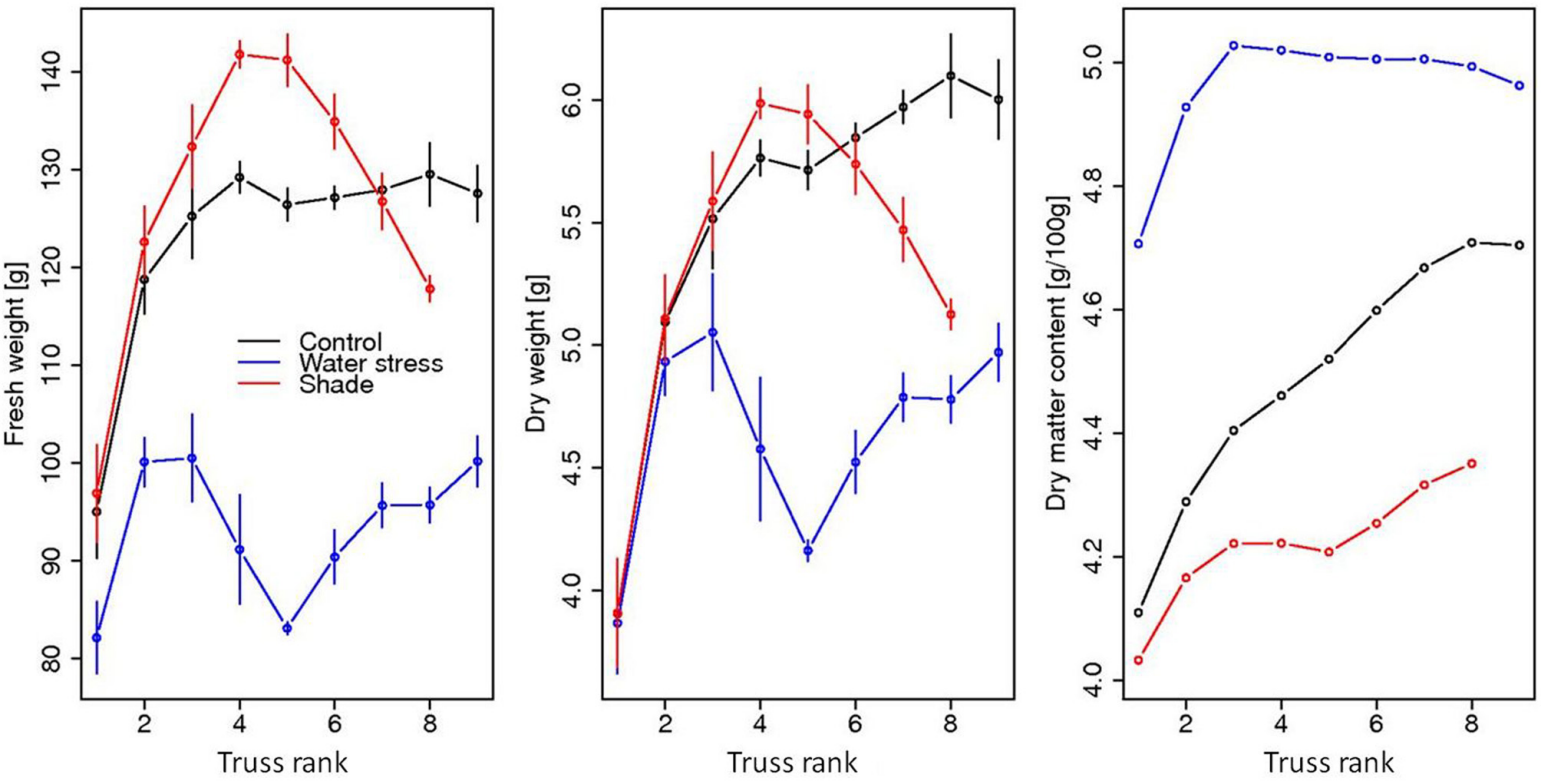
**Evolution, at harvest, of the mean and standard deviation of the simulated fruit fresh masses, dry masses, and dry matter contents in relation with truss rank**.

The integrated fruit/plant model predicts a fruit fresh mass of between 90 and 130 g and a dry mass of about 4–6 g at harvest, depending on the position on the plant (Figure 4, black line). For the 1st trusses in particular, the fruits are smaller compared to later in the season because the leaf area is not totally developed, inducing a shortage in carbohydrate supply. Furthermore, proximal fruits within the trusses are bigger, in relation with the progressive decrease in water potential along the truss (data not shown). A gradual and progressive increase in fruit dry matter content is predicted over time, due to the increase in dry mass rather than to a decrease in fresh mass. All these patterns are consistent with literature data (see Heuvelink review, 2005).

### Symplasmic carbon transport is important for early fruit development

**Figure 5:**
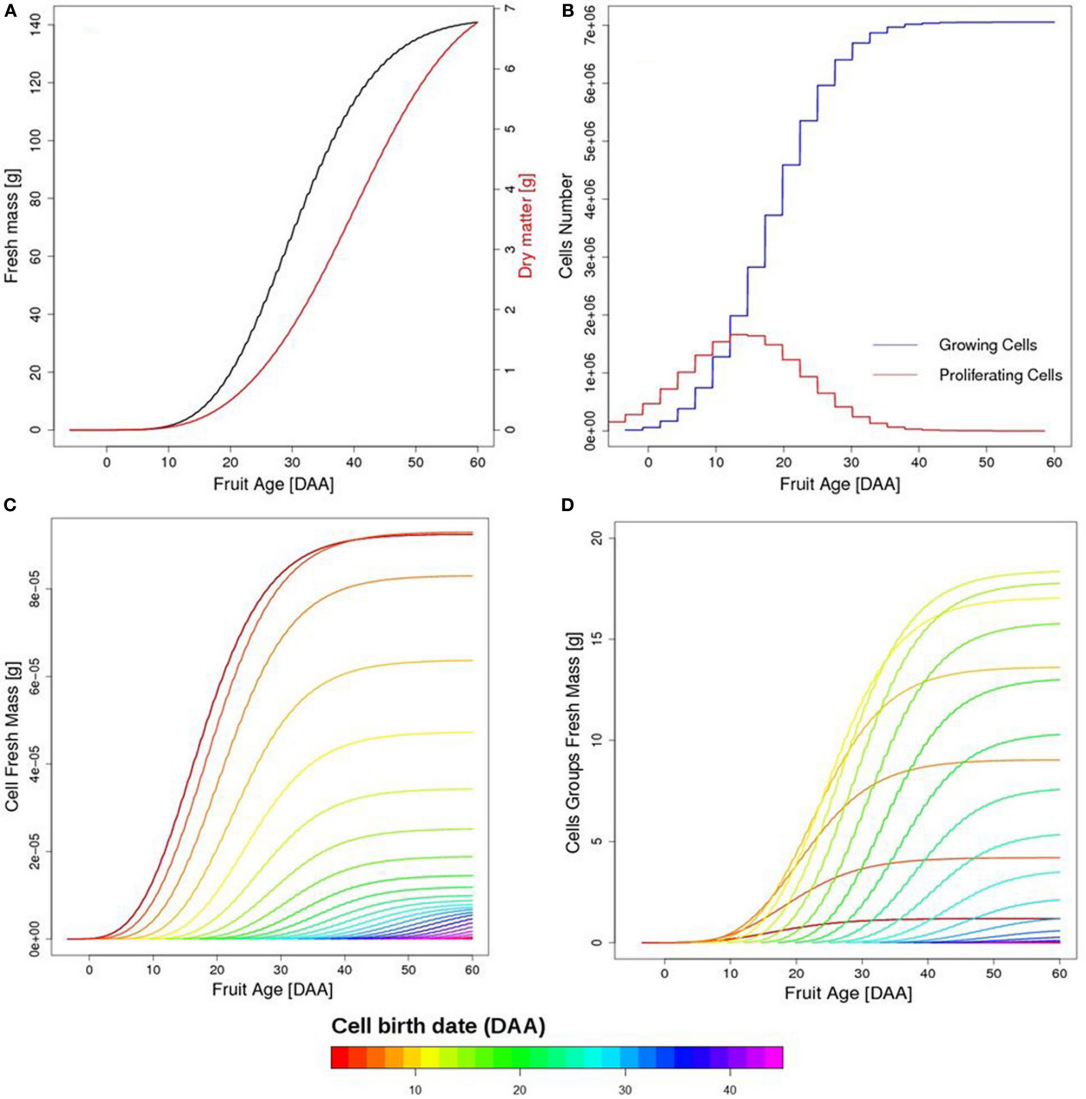
**Temporal evolution of simulated fruit fresh and dry mass (A), as a function of fruit age in days after anthesis (DAA)**. Temporal evolution of growing and proliferating cell populations **(B)**. Growth of individual cells as a function of fruit age (1 cell per group), with colors referring to cell birth date **(C)**. Fresh mass of cell groups as a function of fruit age **(D)**. First born cells reach a higher fresh mass but cells born between 10 and 20 DAA are the most important on the fruit scale on account of their number.

On the fruit scale, fruit growth has a typical sigmoid shape with a rapid increase in fruit mass between 15 and 30 days after anthesis (DAA), in correspondence with the maximum proliferating activity (Figures 5A,B). A progressive slow-down of the growth rate is observed after 40 DAA once cell division has stopped. The main contribution to fruit fresh mass comes from cells born between 10 and 30 DAA, on account of their number, although individual cell growth is higher for cells born in the earliest days of fruit life (Figures 5C,D).

**Figure 6:**
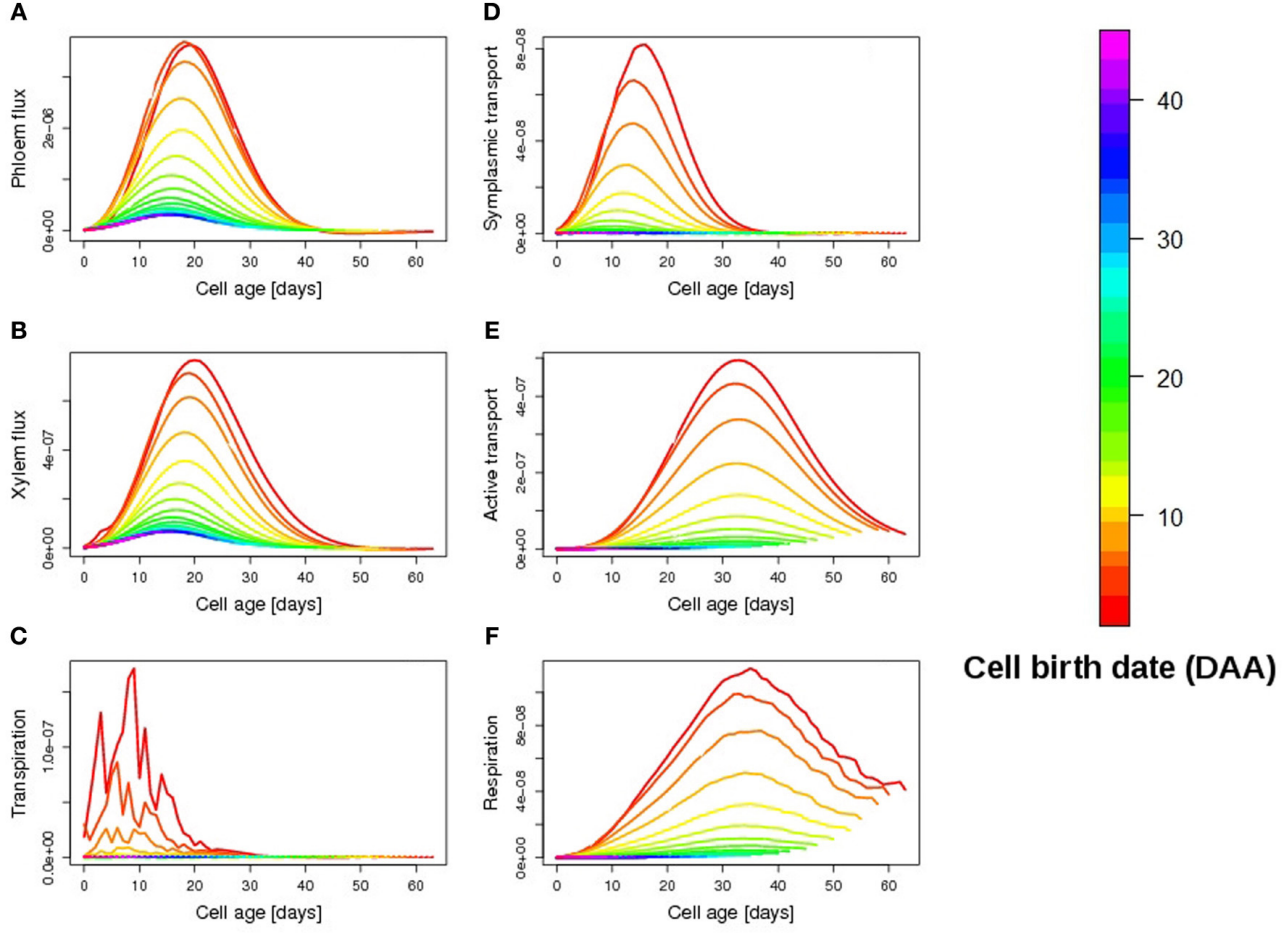
**Temporal evolution of simulated cellular fluxes (daily mean) as a function of cell age: phloem flux (A), (g water/h), xylem flux (B), (g water/h), transpiration (C), (g water/h), symplasmic transport (D), (g sucrose/h), active transport (E), (g sucrose/h), respiration (F), (g sucrose/h)**. The colors refer to cell birth date, red being the first-born cells, a few days before anthesis.

A closer look at individual cell fluxes shows that first-born cells have a higher carbon accumulation rate thanks to the contribution of symplasmic transport during the first days of fruit development (Figure 6D). Although small in magnitude, symplasmic transport actually acts as a starter for the more efficient active transport of carbon, which in turn ensures an exponential increase in dry mass throughout the expansion phase (Figure 6E). The explanation lies in the mathematical form of active transport kinetics which assumes a maximal transport activity proportional to cell dry mass (see Materials and Methods section). During the first days of cell development, therefore, when the dry mass of the cell is very small, symplasmic transport, which depends only on turgor pressure gradient, proves to be the dominant uptake mechanism of the cell. In this way carbon is gradually imported into the fruit, triggering the active transport process once the accumulation of dry mass becomes sufficiently high.

As the fruit respiration is low (Figure 6F), the elevated solute accumulation in early-born cells then induces an increase in both phloem and xylem import from the plant (Figures 6A,B), promoting water accumulation, in spite of the high transpiration rate (high skin conductance ρ, Figure 6C).

Carbon transport by plasmodesmata thus appears to be an effective mechanism to support initial cell growth, promoting a fast carbon and water import into the cell. Indeed, in absence of carbon import by symplasmic transport (e.g., for late-born cells), dry mass accumulation via active transport only, proves extremely delayed in time, the uptake by active transport being low for up to 10 days (Figure 6E). Notice that such a link among symplasmic and active transport processes only depends on the structure of the model and not on the choice of parameter values for the active transport, all within the physiological range.

### Stress response involves changes at the plant and fruit levels

#### Water limitation

The application of the water deficit treatment suddenly decreases plant water potential by around 0.1 MPa, but a gradual recovery is observed later in the season, due to a reduced leaf growth. Since the water deficit is limited, the effect on photosynthesis is essentially linked to the progressive reduction of the leaf surface compared to control—the photosynthesis rate not being impacted. SLA variation was also only limited (Figure 1B). As a consequence, the effect of water deficit on phloem carbohydrate concentration appears reduced and delayed in time. The model indeed predicts a slight increase in phloem concentration until 100 DAS, followed by a rapid fall, up to −10%, (Figure 3D).

**Figure 7:**
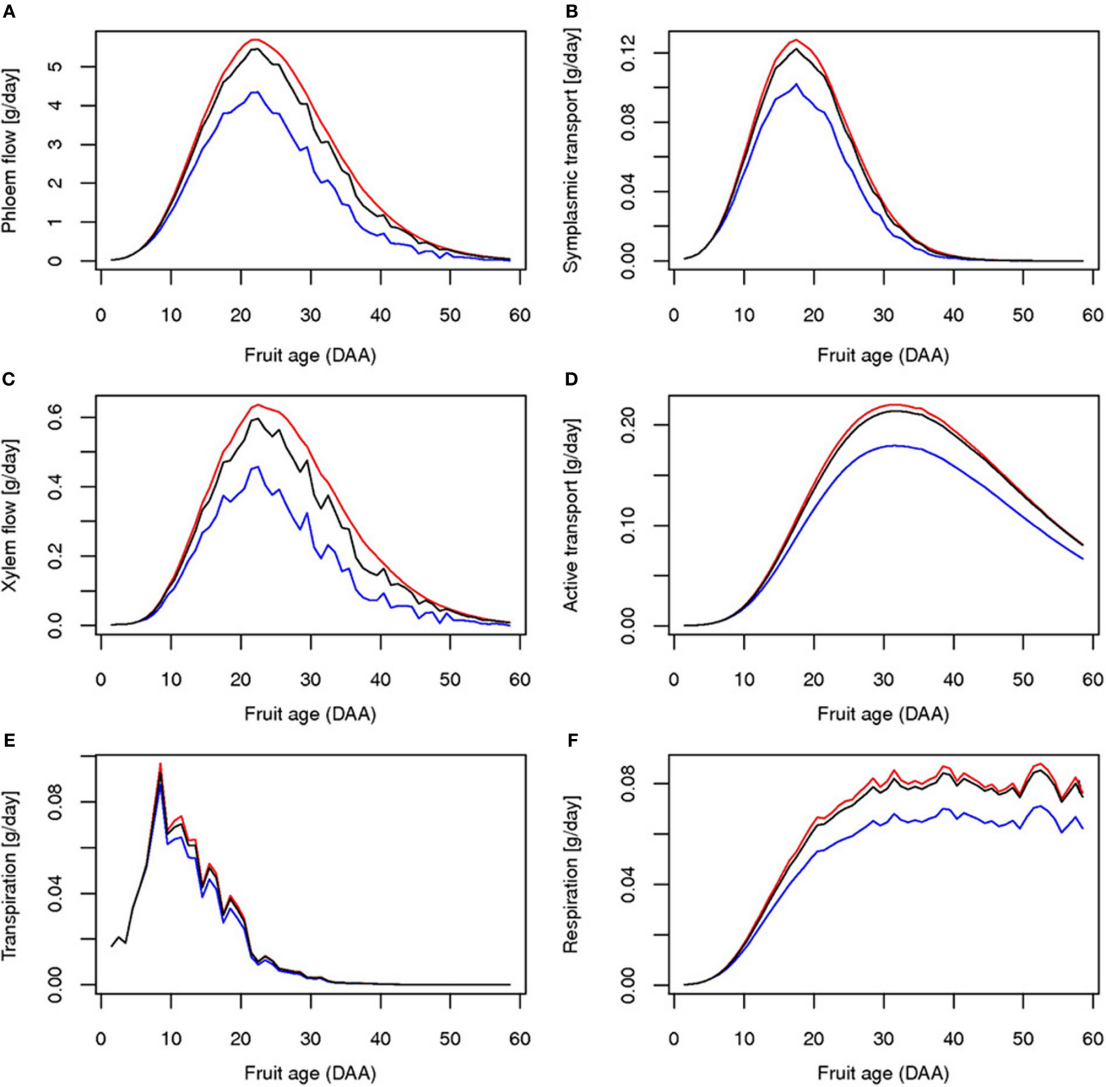
**Simulated daily fluxes (g/h) at the fruit level for truss rank 4 for control (black line), water deficit (blue line), and carbon limitation (red line): phloem flow (A), xylem flow (B), transpiration (C), symplasmic transport (D), active transport (E) and respiration (F)**.

The reduction in resource availability obviously affects fruit growth. As expected, water deficit has a large effect on fruit fresh mass due to the reduction of both phloem and xylem import rates (Figure 7), leading to a weight loss ranging from 16 to 36%. Fruit dry mass is also reduced. The global yield is consequently decreased for fresh and dry matter (Table 1). The decrease in phloem flux, indeed, immediately affects the uptake of carbon by symplasmic transport which leads, on a longer term, to a concomitant reduction of the active transport rate (Figure 7).

**Table 1:** **Predicted effect compared to control at the end of the simulation (160 days) on total yield (fresh, dry weight) and mean fruit dry content, in case of the application of a long stress treatment (water deficit, shade)**.

	**Fruit fresh yield (%)**	**Fruit dry yield (%)**	**Mean fruit dry matter content (%)**
Water deficit	−25	−17	+10
Carbon limitation	−10	−15	−7

#### Carbon limitation

The application of a shade treatment is characterized by important changes at the plant level, both in architecture, resource acquisition, and allocation. Reduction of the incoming radiation causes a drastic drop in the rate of photosynthesis up to half of its control value, with important consequences on plant daily photosynthesis. Faced with such a carbon shortage, the plant reacts with a 2-step strategy. As a first step, the plant reduces its vegetative demand by decreasing both the leaf growth rate and the leaf emergence (Figure 3A). The phyllochron is temporarily multiplied by 3 compared to control, leading to a large delay in the emergence of the 6th and 7th trusses. Indeed, the 7th truss for the shade treatment appears at the same time as the 8th truss for the control. As a second step, a sharing strategy for C is established. Available stored carbohydrate is rapidly reallocated to all organs (data not shown) and the Specific Leaf Area is greatly increased (Figure 1B). Such variation in the SLA could be associated either to a decrease in the dry matter export to the growing leaf or to an increase in leaf growth dimension thanks to the increased water potential (Figure 3C), eventually resulting in larger leaves. This 2-step strategy leads to a modification in the C use by the plant, and allows for a large compensation in leaf growth, ultimately resulting in similar leaf areas for the control and the shaded plant. As a result of such plant plasticity, phloem carbohydrates concentration is only partially affected by the shade treatment, resulting in a maximal loss of 6% with respect to control (Figure 3D). On the fruit scale, indeed, the impact of carbon depletion is quite limited, although the global yield is reduced (Table 1). These results are consistent with previous studies for both vegetative and fruit parts, and for growth and composition as well (Kläring and Krumbein, 2013). For fruits that are already growing at the time of shading application, on the contrary, the model predicts an increase in fruit fresh and dry mass compared to control, which is linked with higher water potential, leading to a transient rise in xylem and phloem flux during carbon limitation (Figure 7).

On the long term, the progressive readjustment of water potential toward the control case, due to the enhanced leaf growth, reduces the “beneficial” effect of carbon depletion, leading to a net weight loss for the last trusses.

### Young cells are more sensitive to stress but recover faster

The effect of carbon depletion and water deficit on cell growth was analyzed as a function of both cell and fruit developmental stages. Indeed, if cell metabolism and wall mechanical properties are linked with cell age, fruit development controls the mechanisms of carbon transport from plant to fruit, providing the available resources for cell growth.

**Figure 8:**
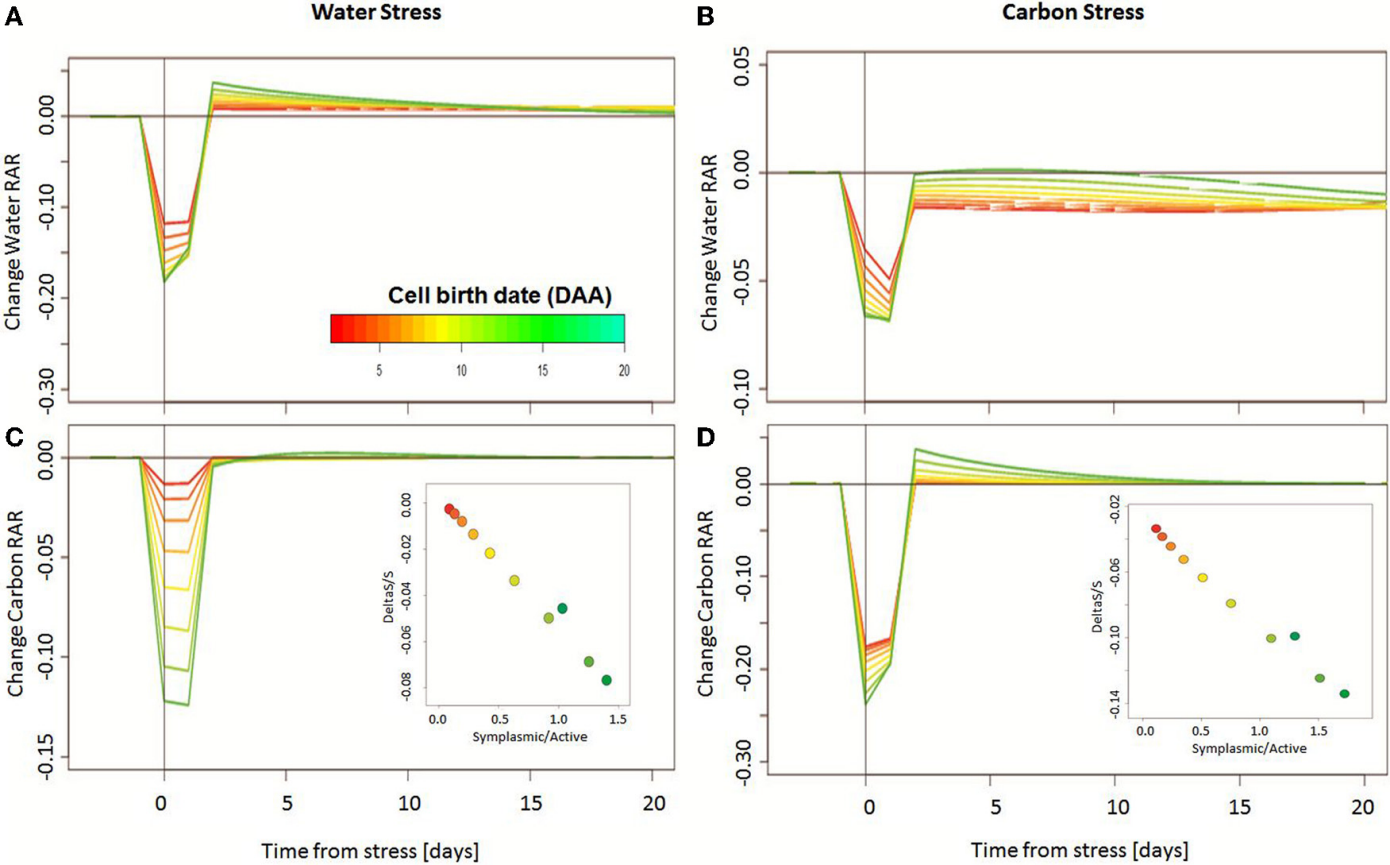
**Change in simulated cell water and carbon relative accumulation rate (RAR) after the application of a 24-h water deficit (A,C) or carbon limitation (B,D)**. Stress is applied at 20 DAA. The colors refer to cell age, red being the oldest and green the youngest, at the time of stress. Inset: following the application of stress, relative dry mass loss is directly linked to the symplasmic over active transport ratio of the cell, at the time of stress. Young cells with a high symplasmic transport appear more sensitive to stress than old ones, with a reduction in dry mass over 5% for water deficit, and over 10% for carbon limitation.

Analysis of stress effects on water and carbon content as a function of cell age shows that young cells are more sensitive to stress, with a strong reduction in their relative accumulation rate (RAR), in both water and carbon, during stress application (Figure 8). Once the stress is released, however, young cells rebound better than old ones, being able to recover and even increase their RAR with respect to control. This is not the case for old cells for which any loss of mass following the application of stress is irreversible, as no significant compensation is observed in their relative accumulation rate after stress release. In extreme cases, the application of carbon limitation to older cells can permanently impair cell growth abilities, resulting in a reduced water accumulation rate even after stress release.

The fast water recovery observed at young ages is essentially linked to the high cell wall plasticity that assures a rapid volume change once water potential is reestablished. For carbon content, the important loss for young cells, as well as their fast recovery time, is intimately related to cell symplasmic-to-active transport ratio, at the time of stress (Figure 8, inset). Indeed, as already mentioned for the control case, carbon transport via plasmodesmata is of major importance to dry mass accumulation during the first days of cell life but it proves more sensitive to both water deficit and carbon limitation, on account of its direct dependence on the plant water and carbon status. As a consequence, the effects of stress application, or of its release, are as strong as this transport mechanism is dominant at the considered time.

### Stress effect depends on fruit age and truss rank

Comparison of the final loss in both fruit fresh and dry mass, shows that the timing of stress application is crucial to determine the overall effect of the treatment at the fruit level (Table 2). The fruit expansion phase (middle stage) turns out to be the most sensitive to stress, with an average water and carbon loss of around 4%, whereas the application of stress in the last part of fruit development results in no or very little effect.

**Table 2:** **Predicted effect on fruit final fresh and dry weight after the application of a short (24 h) stress treatment, with respect to control**.

	**Fruit fresh weight (%)**	**Fruit dry weight (%)**
**WATER DEFICIT**		
Early (5 DAA)	−1.4	−2.8
Middle (20 DAA)	−4.3	−3.1
Late (50 DAA)	−0.6	No effect
**CARBON LIMITATION**		
Early (5 DAA)	−2.1	−4.7
Middle (20 DAA)	−3.6	−6.2
Late (50 DAA)	−0.04	−1.1

*Three modalities for stress application are considered at three different developmental stages*.

In the light of the considerations from the previous section, the greater stress sensitivity observed for fruit in the expansion phase may appear unexpected (Table 2). The young cells and the important symplasmic transport during the early developmental phase could, indeed, suggest an enhanced sensitivity to stress at this time that is progressively reduced as the fruit grows old. The explanation lies in the dynamics of the cell number (Figure 5). Although the effect of stress on individual cells may be strong, the number of cells potentially affected by stress during early development only represents a small fraction of the final cell number in the fruit, thus softening the consequences on the overall mass of the fruit, at maturity. Cell groups born during the expansion phase, instead, are the most numerous ones, so that the application of stress during this phase affects a large number of younger cells, resulting in a marked fruit phenotype. Notice, of course that the above discussion is valid in the case of short, transient stress. In the case of sustained stress, its application from the early developmental phase may be a lot more detrimental for fruit quality than a late one, with possible repercussions on fruit survival too.

At the plant level, indeed, the model shows that the impact of stress strongly depends on the fruit truss rank (Figure 4), but with opposite patterns, depending on the treatment. Interestingly, while the impact of water deficit is immediate and marked, the effects of shade treatment appear delayed, being almost beneficial for trusses from 3 to 6. During the first days after shade application, in fact, fruits can benefit from a higher water potential and still few changes in phloem concentration that both allow for an increased water and carbon accumulation through symplasmic uptake. The beneficial effect, indeed, is enhanced for fruits born in this period (trusses 4 and 5) for which plasmodesmata are fully opened and symplasmic transport is maximal (young fruit age). In the long term, the stabilization of water potential and the decrease in carbohydrate phloem concentration ultimately reduces the uptake of nutrients, resulting in a rapid slowdown in fruit growth for late trusses, in both fresh and dry mass.

In the case of water deficit, a partial recovery in stress tolerance is observed in the long term, thanks to the adaptation of leaf growth. The fruits on the last trusses, indeed, appear less sensitive to water deficit, the impact being maximal for fruits which are setting at the time of stress application. Contrary to the control and shade case, fruit dry content for the water deficit shows a marked increase for the first 3 trusses but remains stable thereafter. Such an effect is linked with the initial reduction in water potential at the fruit insertion point, leading to an important decrease in the xylem and phloem flow toward the fruit. On the long term, the decline in carbohydrate phloem concentration prevents the further increase in dry matter content.

## Conclusions and perspectives

Fruit development and stress response depend on the coordination of several processes, on different scales. Plant architecture, water and radiation availability, rate of transpiration and photosynthesis determine the resources available for fruit development, which in turn affects the overall source-sink balance of the system. In the present paper, the variability of stress effects is analyzed within cell populations, on the fruit scale, and among trusses, at the plant level, for carbon depletion and water deficit.

Results on both scales are in agreement with literature data and confirm a differential effect of water deficit and carbon limitation on the source size and activity, resource allocation, and sink strength. The shade treatment has a strong impact on source activity but not on source size. In our simulations, indeed, the rate of photosynthesis mostly decreased, up to half of control, whereas leaf growth was slightly promoted, supporting the idea of modeling leaf expansion independently of the plant carbon budget (Tardieu et al., 1999). Interestingly, the effect of shade on source activity was partially compensated by an important carbohydrate mobilization from storage compartments, thus limiting the impact of shade on available resources. However, such an effect can possibly be over-estimated in our study due to modeling assumptions, which allow a fast mobilization of carbon reserves.

As in shade, photosynthesis rate and leaf growth behave independently under water limitation, but the effects are opposed. Mild water deficit considerably decreases the leaf expansion rate but does not affect the rate of photosynthesis (see for example review by Muller et al., 2011). The difference in sensitivity between expansive growth and photosynthesis results in a reduced demand but a maintained supply of carbohydrates, leading to increased carbohydrate concentration in plant tissues, as observed in many drought scenarios (Hummel et al., 2010; McDowell, 2011).

Strong differences are predicted between stresses also in what concerns carbon allocation among vegetative and reproductive sinks, giving rise to contrasted fruit phenotypes in both fresh and dry mass.

Whereas water deficit strongly reduces fruit mass, a surprising prediction of the model is indeed the transient increase in fruit fresh and dry mass under carbon limitation, for trusses 3–6. The explanation lies in the dynamics of fruit and leaf responses to the applied treatment.

In leaves, variations in SLA are observed depending on environmental conditions such as radiation, water availability and time of day (Bertin and Gary, 1998). It decreases when environmental conditions have a greater depressive effect on expansion rate than on photosynthesis (Tardieu et al., 1999), as under water deficit, and increases in the opposite case, as in shade.

In our study, a strong increase in SLA is observed during shade treatment, allowing for a larger leaf surface for the same carbon amount. Decreased leaf sink strength then allows for a greater C availability, leading to an increased fruit development. The simulated fruit dry mass would indeed be more reduced (up to 15%) if it is considered that SLA is not impacted by shade.

On the fruit level, an interesting prediction of the model is the importance of symplasmic transport as a catalyst for cell growth, which allows a fast accumulation of osmotically active solutes during the first days of fruit life, when the active transport is not yet effective. An initial sugar accumulation by symplasmic transport in turn promotes the active transport process, further accelerating the import of carbon and water into the fruit, resulting in a positive loop.

During the first days after the application of a shade treatment, a higher plant water potential allows for an increased carbon flow via plasmodesmata, triggering the activation of such a positive loop, and the observed rise in fruit mass for trusses 3–6. Under water deficit conditions, instead, the rapid decrease in plant water potential strongly inhibits the symplasmic transport, resulting in a marked slowdown in the carbon uptake rate and consequent fruit enlargement.

The predicted importance of symplasmic transport on fruit growth is in agreement with its known role in the development and differentiation of new organs (Pfluger and Zambryski, 2001; Zhang et al., 2006; Werner et al., 2011) although the temporal regulation of phloem unloading strategies can vary strongly with the considered species, tissue and developmental stage (Zhang et al., 2004, 2006; Nie et al., 2010; Hu et al., 2011; Werner et al., 2011). In fact, our result may partly be a consequence of some of our modeling assumptions.

The switch from symplasmic to apoplasmic transport as a function of organ age, although in agreement with experimental observations in leaves (Crawford and Zambryski, 2001; Zambryski, 2004), has not been completely proved and other assumptions may be equally valid for fruits. Moreover, the choice of a kinetic rate for the active transport proportional to the previously accumulated dry mass can possibly over-enhance the importance of symplasmic transport at young ages, when dry mass is low.

Unfortunately, current information on phloem transport mechanisms in tomato fruits is still scarce and does not allow a full validation of our modeling hypothesis. In this perspective, the simulation results presented in this paper are thought-provoking and call for renewed attention to the phloem unloading mechanisms and to the role of symplasmic transport during the early phase of tomato fruit development.

In perspective, an improved description of fruit and plant physiology under stress conditions could indeed help to better evaluate the exact contributions of the multiple players of early fruit development. On the modeling side, future challenges include the addition of potential effects of environmental stresses on cell cycle progression (De Veylder et al., 2007; Tardieu et al., 2011; Komaki and Sugimoto, 2012), cell mechanical properties (Cosgrove, 1997), and osmotic regulation (Morgan, 1984; Ashraf and Foolad, 2007). Adjustments in cell cycle duration to environmental conditions, in particular, could partly reduce the current sensitivity of the integrated model to changes in its input variables, by allowing the cells to grow longer in case of nutrient deficiency. The inclusion of fruit photosynthesis as a source of carbohydrates could also be important in sustaining fruit growth during the early developmental stages, when cell division is maximal.

On the plant side, there is a need to better relate the water and assimilate transfer within the plant, especially in order to predict how water deficit affects phloem transport rates and allocation (Lang and Thorpe, 1986; Fisher and Cash-Clark, 2000). Some modeling approaches have already been developed, based on Münch's theory from the 1930s (Lacointe and Minchin, 2008; Jensen et al., 2011; Thorpe et al., 2011; Hall and Minchin, 2013). These studies lay down the theoretical basis of the carbohydrate transfer in the phloem within the plant and enable a full description of the variation in carbohydrate availability within the plant, with possible gradient in the carbohydrate phloem concentration.

In the long run, a better integration between plant and fruit computational models under environmental control, could bring useful insights into the analysis of complex traits, accelerating our capacity to conceive adapted crops and effective cultural practices under stressful conditions (Hammer et al., 2006; Tardieu and Tuberosa, 2010; Yin and Struik, 2010; Ould-Sidi and Lescourret, 2011).

### Conflict of interest statement

The authors declare that the research was conducted in the absence of any commercial or financial relationships that could be construed as a potential conflict of interest.
